# Restoring Biomechanical Gait Function with Ultrasound-Guided Acupotomy for Post-Stroke Equinovarus Foot: Two Case Reports and a Protocol (A CARE- and SPIRIT-Compliant Study)

**DOI:** 10.3390/life15050766

**Published:** 2025-05-10

**Authors:** Jiwoo Kim, Taeseok Ahn, Jihyun Moon, Youngjo So, Hyeon-gyu Cho, Sangho Ji, Myungjin Oh, Sangkwan Lee, Cheol-Hyun Kim

**Affiliations:** 1 Department of Internal Medicine, College of Korean Medicine, Wonkwang University, Iksan 54538, Republic of Korea; wldn3040@naver.com (J.K.); yjyjm98@naver.com (Y.S.); hgyucho@gmail.com (H.-g.C.); jido00atom@gmail.com (S.J.); sklee@wku.ac.kr (S.L.); 2 VARO Korean Medicine Clinic, Seoul 07714, Republic of Korea; truenote87@gmail.com (T.A.); lunarjihyun@gmail.com (J.M.); 3 Department of Acupuncture & Moxibustion Medicine, College of Korean Medicine, Pusan National University, Yangsan 50612, Republic of Korea; kkomc@hanmail.net; 4 Keumkang Korean Medicine Clinic, Cheongju 28145, Republic of Korea; 5 Research Center of Traditional Korean Medicine, Wonkwang University, Iksan 54538, Republic of Korea

**Keywords:** case report, equinovarus foot, protocol, stroke, ultrasound-guided acupotomy

## Abstract

Background: Post-stroke equinovarus foot (EVF) impairs gait stability, increases the risk of secondary injuries, and contributes to elevated healthcare costs. However, effective targeted interventions for EVF remain limited. Patient concerns: Two patients with chronic EVF—a 63.5-year-old male (9.7 months post-stroke) and a 35.7-year-old female (24.5 months post-stroke)—presented with ankle deformity, gait asymmetry, and impaired balance, all of which interfered with daily activities. Intervention and outcomes: Both patients underwent ultrasound-guided acupotomy targeting spastic ankle muscles, administered over four sessions within two weeks. A quantitative gait analysis revealed substantial improvements in step length ratios (Case 1: 0.61 → 0.86; Case 2: 0.67 → 0.88), as well as enhancements in walking velocity, lateral symmetry, postural balance, and Modified Ashworth Scale scores. No adverse events were reported. Protocol proposal: Based on these observations, a prospective randomized controlled trial is planned to compare ultrasound-guided acupotomy plus conventional therapy versus conventional therapy alone. Outcomes will be assessed quantitatively using gait analysis. Lessons and implications: Ultrasound-guided acupotomy may offer a minimally invasive, targeted approach to releasing spastic muscles while preserving neurovascular structures, thereby improving gait function in patients with post-stroke EVF.

## 1. Introduction

Stroke remains a leading cause of mortality and long-term disability worldwide, imposing a profound socioeconomic burden [1]. Among various rehabilitation goals, restoring independent gait is particularly critical, as gait impairments substantially decrease quality of life and elevate the risk of secondary complications, such as deep vein thrombosis, pulmonary embolism, falls, fractures, and pressure ulcers [2]. Effective rehabilitation strategies that promote gait restoration are thus essential for mitigating long-term healthcare costs and enhancing patient outcomes [2].

Lower limb spasticity, a common complication following stroke, has been reported to occur in approximately 25% of all stroke survivors and in nearly 40% of those with accompanying paresis [3]. It plays a pivotal role in disrupting normal gait biomechanics [3]. Equinovarus foot (EVF), a common clinical manifestation of lower limb spasticity, is characterized by excessive ankle plantarflexion and inversion due to the hyperactivity of muscles such as the gastrocnemius, soleus, and tibialis posterior muscles [3]. These mechanical disturbances compromise foot–ground interactions and induce maladaptive changes in pelvic and lumbar kinematics, perpetuating inefficient and unstable gait patterns [4]. Consequently, biomechanical restoration targeting EVF-associated abnormalities is central to optimizing post-stroke rehabilitation [3,4].

Conventional interventions targeting lower limb spasticity, including manifestations such as EVF, often yield suboptimal outcomes [5]. Pharmacological therapies, such as baclofen and tizanidine, are associated with adverse effects, including sedation and cognitive impairment [5]. Although botulinum toxin injections are utilized for focal spasticity management, their efficacy in lower limb applications remains less established, and they carry the risk of exacerbating gait dysfunction through excessive muscle weakening [5]. In clinical settings where concerns regarding muscle strength preservation are prioritized, particularly in ambulatory stroke patients, such risks often lead to a cautious or delayed use of botulinum toxin in the lower limbs [5]. These limitations underscore the need for alternative therapeutic strategies that directly address the biomechanical deficits underlying post-stroke gait impairments.

Ultrasound-guided acupotomy has emerged as a promising modality for biomechanical rehabilitation in post-stroke EVF [6]. By combining the principles of acupuncture with minimally invasive surgical techniques, acupotomy enables the targeted dissection of pathological adhesions within muscle and connective tissue, promoting the recovery of muscle elasticity and joint function [6]. The integration of ultrasound guidance enhances procedural precision, ensuring the safe navigation of anatomical structures and minimizing the risk of iatrogenic injury [7]. Beyond merely reducing spasticity, ultrasound-guided acupotomy holds potential for directly improving gait biomechanics by restoring appropriate muscle–tendon dynamics and enhancing lower limb co-ordination [7]. Moreover, since conventional spasticity scales such as the Modified Ashworth Scale (MAS) often fail to capture dynamic changes in motor function or biomechanical improvements [5], this study prioritized objective gait and balance variables—such as the step length ratio and center-of-pressure (COP) metrics—as more sensitive and functionally relevant outcome measures.

Building upon this rationale, we report two cases of post-stroke EVF treated using a standardized ultrasound-guided acupotomy protocol, with a specific focus on biomechanical gait improvements. To the best of our knowledge, no studies to date have evaluated the effect of ultrasound-guided acupotomy on gait improvement in chronic post-stroke patients using an objective gait analysis, making this report a novel contribution to the field. Quantitative gait analyses were performed to assess changes in the step length ratio, walking velocity, COP area, and COP average velocity, variables that reflect dynamic balance, gait symmetry, and propulsion efficiency. In addition to these exploratory observations, we also present a structured clinical research protocol aimed at validating the biomechanical efficacy of ultrasound-guided acupotomy through a forthcoming prospective clinical trial. This effort may contribute to the development of evidence-based biomechanical rehabilitation strategies for post-stroke gait dysfunction.

## 2. Case Presentation

Among patients with chronic post-stroke EVF treated with ultrasound-guided acupotomy at the authors’ institution, two cases were selected because they underwent full pre- and post-treatment gait assessments and had sufficient clinical documentation to allow for detailed reporting. Importantly, during the treatment period, no other concurrent interventions, such as conventional rehabilitation or Korean medicine therapies, were provided; ultrasound-guided acupotomy was administered as the sole therapeutic intervention. Both cases also completed all planned sessions without missing data, allowing for a consistent and reliable analysis of treatment outcomes.

### 2.1. Case 1

Case 1 involved a 63.5-year-old male patient who had experienced an intracerebral hemorrhage in the right hemisphere, including the basal ganglia, approximately 9.7 months prior, and subsequently underwent conventional rehabilitation. Although he was able to ambulate independently, he demonstrated pronounced gait asymmetry due to left hemiparesis and EVF, which contributed to frequent falls. Despite continuous rehabilitation efforts, no notable improvement in the equinovarus deformity was observed, leading him to seek a traditional Korean medicine intervention at the authors’ institution (Wonkwang University Gwangju Medical Center, WUGMC, Gwangju, Republic of Korea). His current medications are listed in Table 1.

Over a two-week period, the patient underwent four sessions of ultrasound-guided acupotomy targeting the medial and lateral heads of the gastrocnemius, soleus, and tibialis posterior muscles.

Before proceeding with treatment, the patient received a thorough explanation of the procedure, including its potential risks and benefits, and he provided verbal consent to undergo the intervention for therapeutic purposes. A standard safety screening was then conducted to evaluate whether the patient had any medical or procedural conditions that could increase the risk of adverse effects. This included a review of the patient’s history of bleeding tendency, cognitive or psychiatric issues affecting procedural cooperation, use of anticoagulants, local skin problems at the treatment site, immune suppression, or severe anxiety regarding needling procedures. No relevant risk factors were identified in this patient.

**Figure 1:**
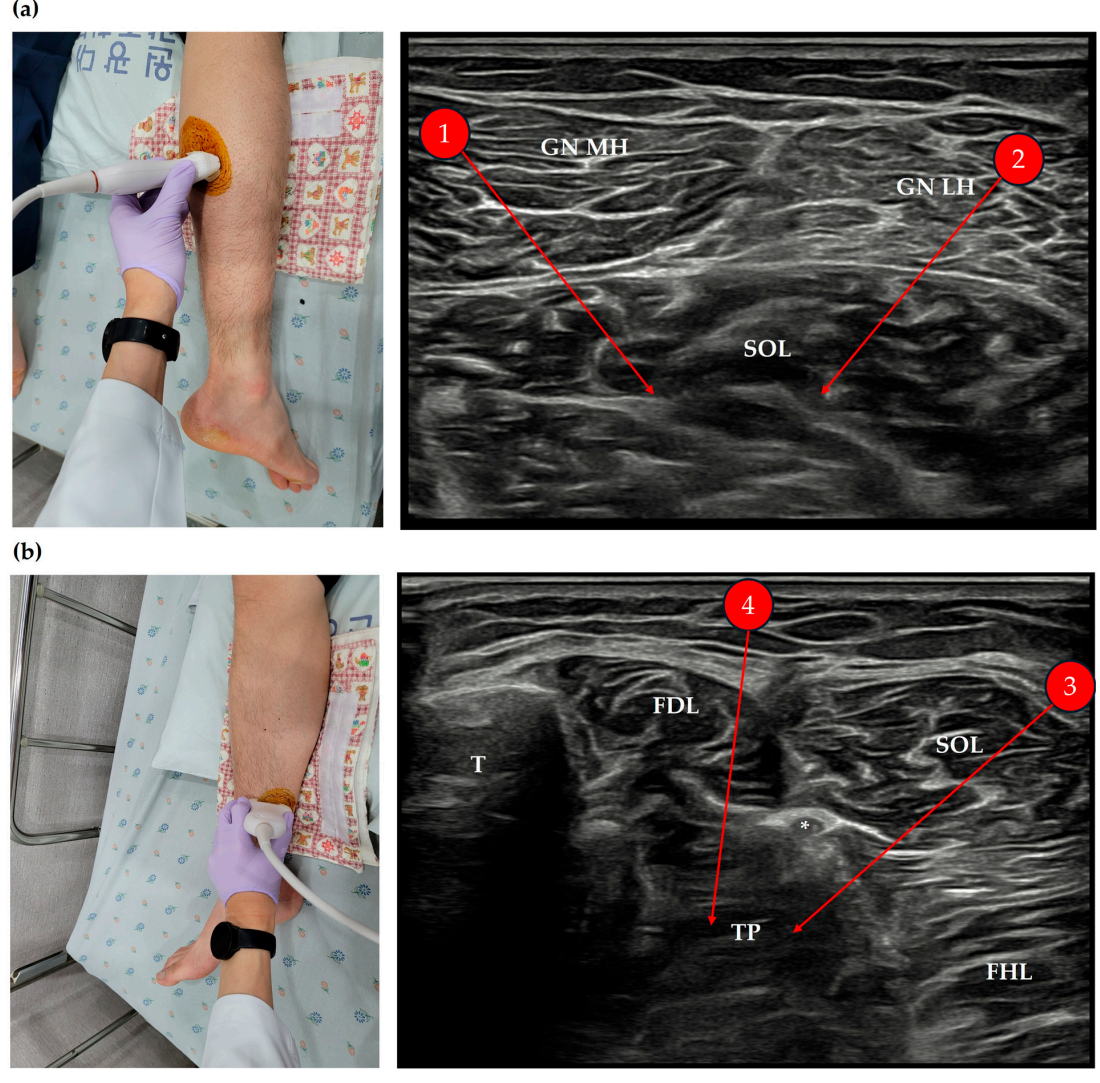
Clinical photographs and corresponding ultrasound images demonstrating the ultrasound-guided acupotomy procedures targeting the medial and lateral heads of the gastrocnemius, soleus (**a**), and tibialis posterior (**b**) muscles. In each panel, the left image shows the probe placement on the patient’s lower limb, and the right image presents the transverse ultrasound scan obtained using an in-plane approach. During the procedures, the acupotomy needle was inserted parallel to the orientation of the muscle fibers and manipulated up to three times at each insertion site. (**a**) Pathways 1 and 2 represent the insertion trajectories targeting the medial and lateral gastrocnemius heads (GN MHs and GN LHs) and the soleus (SOL) muscle, respectively. (**b**) Pathways 3 and 4 depict the insertion pathways through the soleus (SOL) muscle to access the tibialis posterior (TP) muscle. The choice of insertion pathway was determined based on the relative location of the neurovascular bundle (*), ensuring safe needle placement during the procedure. GN MH, gastrocnemius medial head; GN LH, gastrocnemius lateral head; SOL, soleus muscle; FDL, flexor digitorum longus; FHL, flexor hallucis longus; T, tibia; TP, tibialis posterior; *, neurovascular bundle.

The patient underwent ultrasound-guided acupotomy targeting the medial and lateral heads of the gastrocnemius, soleus, and tibialis posterior muscles. The procedures were conducted under sterile conditions using ultrasound-guided in-plane techniques and standardized positioning. A detailed description of the procedural methodology is provided in Figure 1 and in Section 4.4.2 (Ultrasound-Guided Acupotomy) of the study protocol. No adverse events were observed during or after the treatment sessions.

**Figure 2:**
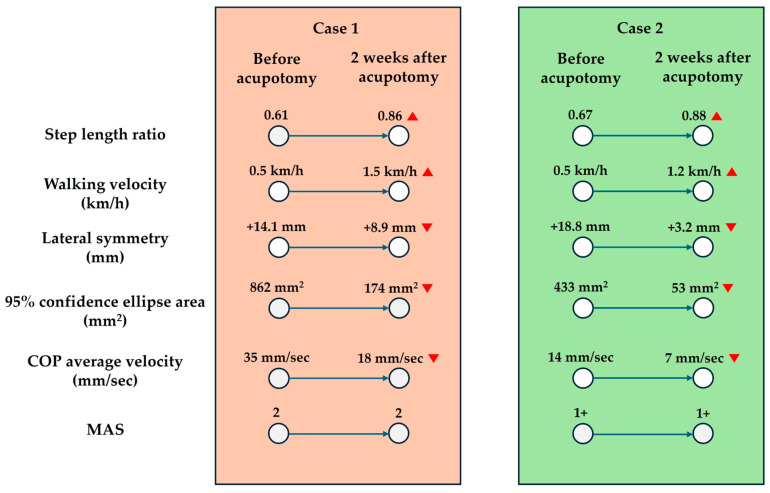
Changes in gait, balance, and spasticity variables before and after ultrasound-guided acupotomy in two stroke patients. While improvements in gait symmetry and balance during standing were observed, no significant differences in the Modified Ashworth Scale (MAS) scores were noted for either case. To facilitate interpretation, the larger value was consistently used as the denominator when calculating the step length ratio. For lateral symmetry, values are expressed as positive (+) when the center of pressure (COP) shifted toward the unaffected side and as negative (–) when shifted toward the affected side, using the midline as the reference. Red arrows (▲ and ▼) indicate increases and decreases in values, respectively. These arrows do not imply clinical improvement or deterioration, as interpretation depends on the specific variable.

Gait, balance, and spasticity were assessed both prior to and following the treatment. Spasticity was measured using the MAS. Gait and balance evaluations were performed utilizing a treadmill system integrated with pressure sensors. For the gait analysis, the patient first walked at a self-selected comfortable speed, determined during an initial familiarization phase, and subsequently walked at that speed for 30 s while the step length ratio and lateral symmetry were recorded. The balance assessment involved the patient standing in a self-selected stable stance width on the treadmill, during which the center-of-pressure (COP) area and average COP velocity were measured over a 10 s period. Explanations of these gait and balance variables are provided in Table 2, and the outcome measurements are illustrated in Figure 2.

**Figure 3:**
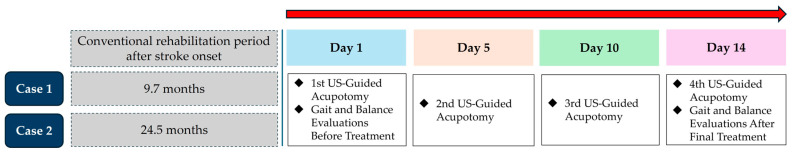
Timeline of post-stroke duration and treatment sessions for the two cases. Cases 1 and 2 had elapsed durations of 9.7 and 24.5 months, respectively, since stroke onset, both exceeding the conventional rehabilitation period. Each patient received four sessions of ultrasound-guided acupotomy on Days 1, 5, 10, and 14. Quantitative gait and balance evaluations were conducted twice: immediately before the first treatment (Day 1) and after the final treatment (Day 14). The red arrow indicates the chronological progression of time across the treatment period. US, ultrasound.

Following ultrasound-guided acupotomy, the patient exhibited improvements in gait and balance. Although no numerical changes were observed in the MAS score, the step length ratio increased, lateral symmetry during walking improved, and balance assessments indicated enhanced postural stability during standing. Figure 3 presents the timeline of the post-stroke duration, treatment sessions, and pre/post-intervention evaluations for both patients, illustrating the clinical context of these functional improvements.

### 2.2. Case 2

Case 2 involved a 35.7-year-old female patient who developed an occlusion of the left middle cerebral artery following an episode of tonsillitis. Mechanical thrombectomy was attempted at the time of ischemic stroke onset but was unsuccessful. Since then, she had undergone conventional rehabilitation for 24.5 months. Although she was able to ambulate independently, she experienced considerable difficulty with turning and exhibited postural instability during walking due to right EVF, leading her to seek treatment at the authors’ institution (WUGMC). Her current medications are listed in Table 1.

Similar to Case 1, the patient received a full explanation of the procedure and provided verbal consent prior to treatment. A standard safety screening was performed and no contraindicating risk factors were identified despite her use of antiplatelet medication. She subsequently received four sessions of ultrasound-guided acupotomy targeting the medial and lateral heads of the gastrocnemius, soleus, and tibialis posterior muscles. The evaluation results are presented in Figure 2, and the clinical timeline is illustrated in Figure 3. Despite being on antiplatelet therapy, no adverse effects were observed during or after treatment. As in Case 1, the MAS score showed no numerical change; however, her step length ratio increased, lateral symmetry during gait improved, and balance assessments indicated enhanced postural stability during standing.

## 3. Patient Perspectives

### 3.1. Case 1

“After my stroke, every hospital I went to told me the same thing–that it would be very difficult to make any further recovery after six months. It was frustrating to hear that over and over again. Even though I could technically walk, I often felt like I would fall with just a small misstep, and I had to be very careful even around the house. Nighttime was especially hard because of severe muscle cramps that often kept me awake. When I asked about possible treatments, the Western medicine doctors explained that since my spasticity was only in the leg, there weren’t effective medications, and that botulinum toxin injections were mainly for arm problems. They didn’t really offer any hope. I felt stuck and helpless. Eventually, I started looking for alternatives myself and learned about acupotomy treatment at a Korean medicine clinic. After receiving it, I felt a noticeable difference–walking became much easier, and best of all, the nighttime cramps disappeared. It was the first time in a long while that I felt hopeful about my recovery.”

### 3.2. Case 2

“When I first told my Western medicine doctor that I was considering acupuncture, he strongly advised against it. Because I was on blood thinners, he warned me that there could be a risk of bleeding and said that acupuncture or similar procedures would not be safe. When I later visited the Korean medicine clinic, I learned that the treatment would involve acupotomy, which uses thicker needles than regular acupuncture. Honestly, I felt even more anxious after hearing that. But when I saw that the entire procedure was performed under ultrasound guidance, it reassured me a lot. It was also much less painful than I had expected. After the first session, I felt some dull soreness for about a day, but it gradually subsided. As the soreness disappeared, I began to notice real changes—my walking became easier, more stable, and smoother. I was no longer afraid of the treatment after that.

“For nearly two years, I had been told that there was no more room for improvement, and I had started to believe it myself. Experiencing these changes after acupotomy felt almost unbelievable. It gave me hope that my body could still recover.

“Looking back, I don’t blame my Western medicine doctor—he was worried about my safety. But I wish there had been a way for doctors from both Western and Korean medicine to talk together and find the best path for me as a patient. If they had worked together, maybe I could have found help sooner. I sincerely hope that in the future, Western and Korean medicine practitioners can cooperate more closely to offer the best possible care for patients like me.”

## 4. Study Protocol

### 4.1. Study Registration

This study protocol was registered with the Clinical Research Information Service (CRIS) of the Korea National Institute of Health (NIH), Republic of Korea (KCT0009924, date of registration: 13 November 2024).

### 4.2. Study Design

#### 4.2.1. Objectives

The primary objective of this study is to evaluate the effectiveness of ultrasound-guided acupotomy in patients with post-stroke equinovarus foot by quantitatively measuring gait variables following the intervention. The secondary objective of this study is to assess the safety of ultrasound-guided acupotomy by monitoring for adverse events, such as local infections, excessive bleeding, or hematoma formation, after the procedure.

#### 4.2.2. Study Period

The study period will be from 1 June 2025 to 31 December 2026.

#### 4.2.3. Name and Address of the Study Institute

Wonkwang University Gwangju Medical Center, 1140-23, Hoejae-ro, Nam-gu, Gwangju, Republic of Korea, 61729.

#### 4.2.4. Name and Title of the Principal Investigator

Cheol-hyun Kim, Assistant professor of Wonkwang University.

#### 4.2.5. Sample Size

- Target number of participants:
  A total of 48 participants (24 in the experimental group and 24 in the control group) are planned to be enrolled.
- Calculation basis:
  This study will be an exploratory clinical trial aiming to evaluate the effects of ultrasound-guided acupotomy on improving gait function in patients with post-stroke equinovarus foot. Exploratory studies of this nature often recommend a minimum number of participants for feasibility. To calculate the target number of participants, the primary efficacy endpoint was set to a significance level (α) of 5% and a power (1 − β) of 80%. The efficacy variable selected was the step length ratio, as referenced in a previous study [9]. Due to the scarcity of studies using ultrasound-guided acupotomy for post-stroke equinovarus foot, we referred to studies that measured the step length ratio as an efficacy indicator. A study by Patterson et al. [9] compared healthy individuals and patients with stroke, demonstrating a significant difference in the step length ratio before and after treatment.
  The step length ratio approaches 1 as gait symmetry improves, and it decreases as asymmetry increases. In Patterson et al.’s study [9], the step length ratio was 0.87 in the stroke group and 0.97 in the healthy group, with a statistically significant difference at a significance level of 0.01. Based on these data, we assumed a clinically meaningful change in the step length ratio (ε) of 0.10 and a common standard deviation (δ) of 0.11. Using these variables, the calculated sample size per group was 19. Based on an expected dropout rate of 20%, the sample size per group was adjusted to 24, resulting in a total of 48 participants for the study.

#### 4.2.6. Study Flow Diagram

**Figure 4:**
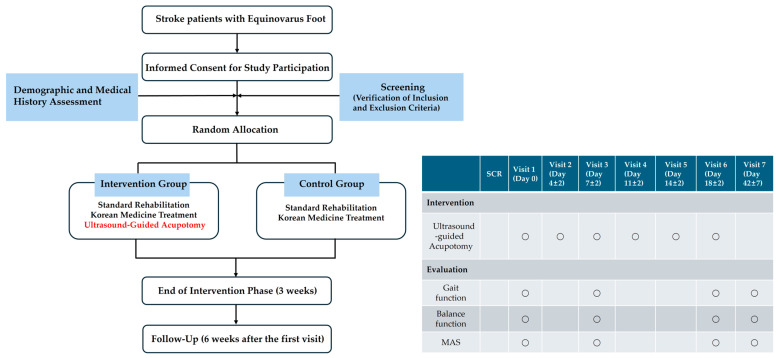
Study flow diagram. SCR, screening. Arrows indicate the chronological flow of the study procedures. Text in red highlights interventions that were administered exclusively to the intervention group and not to the control group. Circles indicate that the corresponding intervention or evaluation was performed on that specific visit day.

The study flow diagram is presented in Figure 4.

### 4.3. Participants

#### 4.3.1. Target Disease and Symptoms

Stroke and stroke-related equinovarus foot.

#### 4.3.2. Inclusion Criteria

- Adults aged 19 years or older;
- Patients previously diagnosed with stroke (ischemic stroke, hemorrhagic stroke, or subarachnoid hemorrhage; KCD codes I60-I63) based on clinical presentation and radiological findings;
- Patients who have passed at least 3 months (90 days) since stroke onset;
- Patients presenting with spastic equinovarus foot on the affected side with an MAS score of 2 or higher;
- Patients with a functional ambulation category score of 3 or higher;
- Patients who fully understand the study aim and voluntarily consent to participate by providing written consent either personally or through a guardian/proxy.

#### 4.3.3. Exclusion Criteria

- Patients with traumatic hemorrhagic or subarachnoid hemorrhage;
- Patients with unstable vital signs requiring bed rest following stroke onset;
- Patients who have initiated treatments for spastic equinovarus foot (e.g., botulinum toxin injections, chemical nerve block, surgical interventions, and intrathecal baclofen therapy) within the past 3 months;
- Patients with spasticity caused by congenital deformities or skeletal abnormalities unrelated to stroke;
- Patients on anticoagulant therapy with an INR ≥ 4.0;
- Patients with severe coagulation disorders, such as hemophilia;
- Patients with signs of infection or other skin conditions at the site of acupotomy application;
- Patients deemed physically or mentally unfit for study participation based on clinical judgment.

#### 4.3.4. Allocation Method and Blinding Limitations

Blinding will not be applied in this study because the ultrasound-guided acupotomy procedure requires real-time visualization and precise operator control, making it inherently unsuitable for blinding methods. The randomization method will be as follows:

- Participants enrolled in this clinical study will be assigned to either the experimental or control groups randomly to prevent biases during group allocation.
- A blocked randomization method with a block size of 4 will be used to ensure equal allocation across both groups. Randomization codes will be pregenerated, with the block size set to 4.

The randomization process will be conducted as follows:

- Each participant who consents to join the clinical study will be assigned a screening number in the order in which they provide written consent. If multiple participants are screened on the same day, numbers will be assigned based on the order of consent.
- Participants who meet all inclusion/exclusion criteria will be randomized at visit 1 (Day 0) according to a pregenerated randomization table created by a statistical expert. A randomization number will be assigned to each participant.
- If a participant withdraws from the study, their assigned randomization number cannot be reused, and the withdrawn participant cannot rejoin the study.

#### 4.3.5. Criteria for Discontinuation and Dropout

- Violation of the inclusion or exclusion criteria.
- The occurrence of a serious adverse event or participant’s request to discontinue due to adverse events.
- Withdrawal of consent by the participant or their guardian/proxy.
- The determination that continuing the study is inappropriate by the principal investigator or the study co-ordinator.

### 4.4. Intervention

#### 4.4.1. Standard Rehabilitation and Korean Medicine Treatments

- Both the experimental and control groups will receive standard rehabilitation therapy and Korean medicine treatments for 6 weeks.
- Rehabilitation and Korean medicine treatments refer to commonly practiced therapies for patients with stroke and must not include any additional interventions specifically targeting lower limb spasticity relief.
- Each standard rehabilitation therapy session will comprise both physical therapy (functional electrical stimulation therapy, neurorehabilitation therapy, and gait training) and occupational therapy (complex occupational therapy and daily living activities). Each session will last for 1.5 h, with 5–10 sessions per week.
- Korean medicine treatments will consist of manual acupuncture therapy, electroacupuncture therapy, and herbal medicine therapy. Manual acupuncture and electroacupuncture treatments will be administered once daily, with seven sessions per week, while herbal medicine will be taken daily at doses of 2–3 packs per day (110 cc per pack).

#### 4.4.2. Ultrasound-Guided Acupotomy

**Figure 5:**
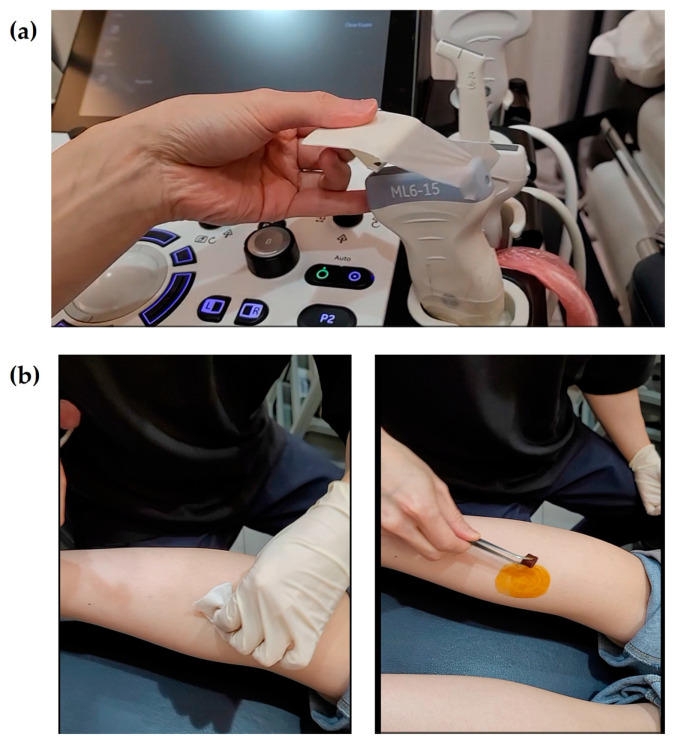
Procedures for infection prevention. To prevent infection, a sterile disposable surgical contact drape (3M Steri-Drape^TM^, model 1050, St. Paul, MN, USA) was applied to the ultrasound probe prior to the procedure (**a**). The treatment area was then disinfected twice: first with an alcohol swab (**left image**) and then with povidone–iodine (**right image**) (**b**).

- The experimental group will receive ultrasound-guided acupotomy in addition to standard rehabilitation and Korean medicine treatments, administered six times over three weeks.
- The acupotomy needles will have the following dimensions: 0.5 mm × 80 mm.
- Before the procedure, a sterile drape (disposable surgical contact drape, 3M Steri Drape 1050) will be applied to the ultrasound probe (Figure 5a). The probe and the treatment site will then be disinfected twice using an alcohol swab and povidone–iodine solution (Figure 5b).
- The treatment will target three regions and four specific muscles: the medial and lateral heads of the gastrocnemius, the muscle belly of the soleus, and the muscle belly of the tibialis posterior. Using an in-plane ultrasound-guided technique, the gastrocnemius and soleus muscles will be treated with the patient in a prone position (Figure 1a). Two insertion pathways will be used to sequentially target both gastrocnemius heads and the underlying soleus muscle, enabling effective stimulation through shared access points. The tibialis posterior muscle will be treated in the supine position, with the affected limb externally rotated approximately 30°, using a medial approach along the tibia (Figure 1b). The precise scanning points will be determined based on the tenderness points in each muscle. If tenderness points are difficult to identify due to sensory loss, the treatment site will be selected based on anatomical landmarks and the region of maximal muscle thickness. For the tibialis posterior muscle, scanning will focus on avoiding major blood vessels and nerves. At each insertion point, the acupotomy needle will be manipulated up to three times to elicit a deqi sensation, which will be verbally confirmed by the patient whenever possible.
- After the acupotomy procedure, the treated area will be disinfected with an alcohol swab, followed by cupping therapy for five minutes. The intensity of cupping will be minimized to prevent excessive muscle relaxation, and the cups will be immediately removed once bleeding has ceased at the treatment site. The area will then be disinfected again with an alcohol swab, and a sterile circular adhesive bandage will be applied.
- Post-procedure reactions, such as bruising, pain, or minor bleeding, will be carefully monitored. To prevent dizziness-related falls, patients will be advised not to stand immediately after the procedure. Instead, they will be encouraged to remain seated until their body has fully adjusted. After standing, patients will be closely observed for any signs of dizziness or instability, and movement will be restricted until sufficient stabilization is ensured.

#### 4.4.3. Prohibited Concomitant Medications and Pharmacological Treatments

During the study period, no treatments specifically aiming to alleviate equinovarus foot should be administered, except for standard rehabilitation therapy, Korean medicine treatments, and ultrasound-guided acupotomy. However, treatments intended to alleviate equinovarus foot that have been consistently administered for at least three months prior to screening may be continued.

### 4.5. Outcomes

To minimize detection bias, all gait and balance variables will be assessed by a third-party evaluator who is not involved in treatment administration or group assignment.

#### 4.5.1. Primary Efficacy Outcome

- Step length ratio
  Step length is defined as the distance from the heel of one foot to the heel of the opposite foot. According to Patterson et al. [9], the step length ratio is one of the most useful gait variables for evaluating walking in patients with stroke. The step length ratio is calculated by dividing the step length of the affected side by that of the unaffected side to facilitate interpretation. For example, a step length ratio of 0.5 indicates that the step length on the unaffected side is twice as long as that on the affected side. A step length ratio closer to 1 indicates a gait pattern more similar to that of a healthy individual [9].

#### 4.5.2. Secondary Efficacy Outcomes

- Gait function
  ○ Walking velocity (km/h):
    Walking velocity is an essential indicator reflecting walking ability in patients with stroke [10]. An increase in walking velocity is associated with an improved quality of life in patients with stroke [10].
  ○ Lateral symmetry (mm):
    Lateral symmetry refers to the side-to-side movement of the center of pressure (COP) during walking [11]. A larger positive value indicates a shift of the COP toward the right side, while a larger negative value indicates a shift toward the left side [11]. In this study, to ensure ease of interpretation, the unaffected and affected sides will be consistently denoted with positive (+) and negative (–) values.
- Balance function
  ○ 95% confidence ellipse area (mm^2^):
    This variable represents the range of the COP movement path during standing [11]. A larger area indicates greater body sway, suggesting that the COP must move across a wider range to maintain balance [11]. Consequently, a larger ellipse area reflects postural instability.
  ○ COP average velocity (mm/s):
    This variable indicates the average speed of COP movement during standing [11]. A higher COP average velocity indicates faster movement of the COP, indicating either postural instability or an increased effort to maintain balance [11]. Consequently, a higher velocity reflects postural instability.
- Modified Ashworth Scale (MAS)
  The MAS is a clinical scale used to assess muscle tone by evaluating the resistance encountered during passive joint movement [12]. This assessment does not require specialized equipment and can be performed quickly.

#### 4.5.3. Safety Assessment

- Safety outcomes will be evaluated by assessing reactions observed following ultrasound-guided acupotomy treatment. These reactions will be categorized into bruising, pain, microbleeding, extensive bleeding, hematoma, local infection, edema, fatigue, autonomic nervous system dysfunction, and others. The frequency of each reaction will then be analyzed.

#### 4.5.4. Time Points for Assessment and Data Collection Methods

- The assessment time points are illustrated in Figure 4. The step length ratio, walking velocity, and lateral symmetry will be measured using a treadmill with an embedded force plate (FDM-T, Zebris Co., Ltd., Germany). Participants will walk on the treadmill at their most comfortable speed for 30 s, during which the above variable will be automatically recorded. The 95% confidence ellipse area and COP average velocity will be measured while the participant stands still on the same treadmill for 10 s. The MAS will be assessed by a trained Korean medicine practitioner, who will passively move the participant’s ankle joint to evaluate the degree of resistance in the muscles.

### 4.6. Statistical Analysis

#### 4.6.1. General Principles

- The stroke data obtained from the participants in this clinical study will be analyzed using both the intention-to-treat (ITT) and per-protocol (PP) methods. The primary analysis will be conducted using the ITT approach, while the PP analysis will be performed separately to ensure consistency with the ITT results. All statistical analyses will be two-tailed, with a significance level of 5%. Descriptive statistics for demographic, sociological, and baseline characteristics will be presented for each group.
- For continuous data, independent *t*-tests or Mann–Whitney U tests will be used, depending on the normality of the data distribution. Categorical data will be analyzed using the Chi-square test or Fisher’s exact test. If significant differences are found in the baseline characteristics between the two groups, differences will be adjusted during the efficacy analysis to account for heterogeneity. Statistical analyses will be performed using SPSS Version 23.0 (IBM Corp., Armonk, NY, USA).

#### 4.6.2. Efficacy Outcome Variables

- For the analysis of efficacy outcome variables, independent *t*-tests will be applied to verify the homogeneity of baseline values between the intervention and control groups, provided that normality is assumed. If homogeneity is confirmed, changes in outcome variables from baseline to the final intervention will be analyzed within each group using paired *t*-tests.
- If there are significant baseline differences between the two groups, an analysis of covariance (ANCOVA) will be performed, using the group as a fixed factor and the baseline values as covariates. If normality is not assumed, a Wilcoxon signed-rank test will be applied to assess within-group changes.

## 5. Discussion

This manuscript was designed with a dual purpose: to report two preliminary cases of post-stroke EVF treated with ultrasound-guided acupotomy and to present a structured clinical research protocol derived from these observations. The integration of clinical findings with protocol development reflects a translational approach that bridges real-world therapeutic outcomes with a systematic investigation.

In both cases, we explored the potential role of ultrasound-guided acupotomy in restoring biomechanical gait function, using an objective gait analysis to assess treatment outcomes. The observed improvements in gait biomechanics served as the foundational basis for developing a formal clinical trial protocol, which is presented in the main body of this manuscript.

Rehabilitation following stroke is traditionally regarded as most effective within the “golden period” of six months post-onset [8]. Both patients in this report had surpassed this window and had been advised that further neurological recovery would be unlikely. Nonetheless, while neuroplasticity peaks within the early months [8], the biomechanical abnormalities contributing to EVF—such as muscle contractures, fibrotic adhesions, and altered tendon structures—remain modifiable beyond this timeframe [13]. Therefore, therapeutic strategies targeting biomechanical dysfunctions may still promote meaningful functional recovery in chronic-stage stroke survivors. Notably, both patients expressed initial discouragement due to having been told that further improvement was improbable. However, they each reported surprise and gratitude upon experiencing meaningful gait and balance recovery. These qualitative perspectives underscore the clinical relevance and patient-centered impact of biomechanical interventions such as ultrasound-guided acupotomy, particularly in populations considered beyond the conventional recovery window.

These observations provide a deeper understanding of the pathophysiology of EVF and the limitations of current treatment strategies, thereby highlighting the potential value of novel biomechanical interventions. EVF is a common clinical manifestation of lower limb spasticity following stroke, resulting primarily from the hyperactivity of muscles such as the gastrocnemius, soleus, and tibialis posterior muscles [14]. This abnormal muscle activity leads to excessive plantarflexion and inversion at the ankle, impairing foot clearance, destabilizing dynamic balance, and contributing to asymmetric gait patterns [15]. Such biomechanical disruptions substantially increase the risk of falls and fractures, compounding the overall burden of stroke-related disability. Despite the clinical significance of EVF, standardized treatment protocols have not been firmly established. Pharmacologic agents such as baclofen and tizanidine, although effective in reducing generalized spasticity, frequently cause side effects, including drowsiness and cognitive dysfunction [5]. Botulinum toxin injections, while beneficial for focal spasticity, may exacerbate muscle weakness in the lower limbs, thereby worsening gait biomechanics [5].

Acupotomy has been proposed as a potential biomechanical intervention that directly targets soft tissue adhesions and muscular architectural abnormalities. Experimental studies suggest that acupotomy may reduce inflammatory cell infiltration, facilitate the reorganization of muscle fibers, and alleviate fibrotic changes within affected tissues [16]. Additionally, acupotomy appears to influence apoptotic signaling pathways, such as Bax and caspase-3 downregulation [17], potentially contributing to the preservation of muscular elasticity and preventing secondary biomechanical deterioration associated with chronic spasticity.

Although acupotomy offers therapeutic potential, its inherent invasiveness necessitates precise techniques to minimize the risk of unintended neurovascular injury. To mitigate these risks, ultrasound-guided methods have been increasingly incorporated into acupotomy procedures. Ultrasound allows for the real-time visualization of soft tissue structures without radiation exposure, thereby enhancing both procedural safety and targeting accuracy [18]. By enabling precise intervention at sites of biomechanical dysfunction, ultrasound-guided acupotomy may contribute to improvements in gait biomechanics.

The two cases presented illustrate different stroke etiologies, one hemorrhagic and one ischemic, with the latter patient maintained on long-term antiplatelet therapy. Despite the theoretical risk of bleeding complications associated with antithrombotic agents, ultrasound guidance allowed the procedures to be performed safely, and no adverse events were observed [13]. These findings suggest the potential clinical feasibility of ultrasound-guided acupotomy, even in patients with complex medical profiles.

A quantitative evaluation is critical for exploring the therapeutic potential of interventions aimed at improving gait biomechanics. While mild-to-moderate lower limb spasticity may provide functional benefits for postural support [5], rehabilitation strategies should focus not only on reducing spasticity but also on optimizing biomechanical gait variables. Prior investigations, such as one by Oh et al. [19], have reported a disconnect between clinical measures of spasticity and actual improvements in gait performance. This complex relationship between spasticity measures and functional outcomes warrants further examination in our study context. In our case observations, gait analysis was performed using a treadmill-based force plate system (FDM-T, Zebris Co., Ltd., Isny, Germany) to assess multidimensional variables closely related to gait biomechanics, including the step length ratio, walking velocity, lateral symmetry, COP area, and COP average velocity. These objective variables may serve as foundational indicators of biomechanical gait function improvements in post-stroke patients.

In both cases, improvements were observed across multiple gait and balance variables following ultrasound-guided acupotomy. Notably, the step length ratio approached values closer to 1.0, suggesting more symmetric gait patterns [9]. Walking velocity increased markedly, indicating enhanced functional mobility [10]. Lateral symmetry values moved closer to zero, indicating reduced side-to-side asymmetry and a more balanced weight distribution between the paretic and nonparetic limbs during gait [11]. Furthermore, reductions in the COP area and average velocity during quiet standing indicated better postural stability and control [11]. Collectively, these multidimensional improvements provide supportive evidence that biomechanical function can be enhanced following acupotomy, even in the absence of substantial changes in spasticity, as measured by the MAS. This observation raises an important question regarding the role of spasticity reduction in the restoration of biomechanical gait function, particularly given the minimal changes in MAS scores noted in both patients.

While both patients showed preliminary improvements in gait variables and balance metrics following ultrasound-guided acupotomy, it is noteworthy that these functional improvements occurred despite minimal changes in MAS scores. This finding warrants further discussion in relation to our hypothesis and the broader understanding of spasticity management in post-stroke rehabilitation.

The minimal changes observed in MAS scores, alongside substantial functional improvements, suggest that the relationship between clinically measured spasticity and functional outcomes may be more complex than traditionally understood. Our observations align with emerging research suggesting that functional improvements in post-stroke patients can occur independently of significant reductions in clinical measures or spasticity [20,21,22]. This phenomenon could be explained by several mechanisms.

First, ultrasound-guided acupotomy may selectively affect the mechanical properties of spastic muscles without necessarily altering the neurological components that contribute to the MAS score. By mechanically releasing adhesions and fibrotic tissues within spastic muscles, the technique may improve muscle extensibility and joint mobility without dramatically changing the velocity-dependent resistance measured by the MAS.

Second, the improvement in gait and balance despite minimal MAS changes supports the hypothesis that functional recovery after stroke depends on multiple factors beyond spasticity reduction alone, including improved proprioception, enhanced motor control, and neuroplastic changes in motor pathways. Acupotomy may influence these factors through mechanisms that are not fully captured by the MAS assessment.

Third, the MAS, while widely used clinically, has known limitations in sensitivity to subtle changes in muscle tone, especially in chronic spasticity where structural changes in muscles have become established [23]. The functional improvements observed may reflect changes in dynamic muscle function during movement that are not adequately captured in the static assessment of the MAS.

This observation has important clinical implications for spasticity management in post-stroke rehabilitation, suggesting that (1) treatments focusing exclusively on reducing clinically measured spasticity may not necessarily translate to functional improvements; (2) functional improvements can occur through mechanisms other than tone reduction; and (3) treatment goals should prioritize functional outcomes rather than isolated spasticity reduction.

Building upon the above observations, several limitations of this study should be acknowledged. First, this report included only two patients, which inherently limits the generalizability of the findings. Nonetheless, it is important to note that both patients, despite being beyond the conventional rehabilitation window, showed measurable improvements in gait biomechanics, as verified through a quantitative analysis. Furthermore, a prospective randomized controlled trial has already been scheduled based on a detailed clinical research protocol developed from these preliminary observations. Such integration of clinical outcomes and structured protocol development within a case report format remains relatively uncommon. Second, due to the procedural nature of ultrasound-guided acupotomy, a double-blind study design could not be implemented. However, ultrasound guidance ensured procedural consistency and objectivity, and, to minimize detection bias, all gait and balance variables in the planned trial will be assessed by a third-party evaluator who is not involved in treatment administration or group assignment. Third, the sample size calculation for the proposed clinical trial was based on secondary data derived from a study involving a different type of intervention. Although the referenced study used the step length ratio as a common efficacy indicator, differences in treatment modality may limit the precision of this estimation. Nevertheless, in the absence of prior studies on ultrasound-guided acupotomy for post-stroke EVF, the use of comparable biomechanical outcome data from related research is a widely accepted approach for estimating sample size in exploratory trials and is considered methodologically appropriate.

This case report provides preliminary evidence suggesting that ultrasound-guided acupotomy may serve as an effective biomechanical intervention for restoring gait function in patients with post-stroke EVF. Based on these initial findings, a clinical research protocol, compliant with SPIRIT guidelines, was developed. A prospective clinical trial, supported by the Ministry of Health and Welfare of the Republic of Korea, is scheduled to commence in June 2025 and is designed to rigorously evaluate the efficacy and safety of ultrasound-guided acupotomy for post-stroke EVF.

## Figures and Tables

**Table 1:** Current medications used in the two cases.

	Generic Names	Dosages and Frequencies
Case 1	Choline alfoscerate 400 mg	3 capsules, three times daily
	Mosapride citrate hydrate 5.29 mg	3 tablets, three times daily
	Acetyl-L-camitine hydrochloride 590 mg	3 tablets, three times daily
	Amlodipine besylate 6.944 mg	1 tablet, once daily
Case 2	Clopidogrel bisulfate 97.875 mg	1 tablet, once daily
	Atorvastatin calcium trihydrate 10.85 mg	1 tablet, once daily

**Table 2:** Variables obtained using the treadmill with an embedded force plate (FDM-T, Zebris Co., Ltd., Isny, Germany).

Variables	Explanation
Step length ratio	Step length represents the linear distance between the heel contacts of opposite feet during successive steps [8]. In patients with gait impairments, a reduced step length is often observed, reflecting compromised gait stability and increased energy demands [8]. As noted by Patterson et al. [9], step length is a critical variable for assessing gait symmetry after stroke, with the step length ratio providing a simplified metric; values closer to 1 suggest more symmetric gait patterns.
Walking velocity	Walking velocity, defined as the speed of ambulation over a given distance, is a primary indicator of functional recovery in individuals post-stroke [10]. Increases in walking velocity are associated with better rehabilitation outcomes and overall improvements in quality of life [10].
Lateral symmetry	Lateral symmetry quantifies the medial–lateral displacement of the center of pressure (COP) during gait. Larger positive or negative values indicate greater deviations toward the right or left side, respectively [11]. In this study, shifts toward the nonparetic side were recorded as positive, while shifts toward the paretic side were recorded as negative, relative to the midline. A value approaching zero signifies improved lateral balance and gait symmetry [11].
95% confidence ellipse area	The 95% confidence ellipse area of the COP measures the dispersion of postural sway during quiet standing [11]. It represents the smallest ellipse encompassing 95% of COP trajectories [11]. A smaller ellipse area corresponds to enhanced postural stability and reduced sway amplitude [11].
COP average velocity	COP average velocity describes the mean speed of center-of-pressure movement during a standing task [11]. Lower COP average velocities reflect greater postural control, indicating reduced instability and more efficient balance maintenance [11].

## Data Availability

The data supporting the findings of this study are available from the corresponding author upon reasonable request.

## Abbreviations

The following abbreviations are used in this manuscript:

ANCOVA	analysis of covariance
COP	center of pressure
CRIS	Clinical Research Information Service
EVF	equinovarus foot
FAC	functional ambulation category
ITT	intention to treat
KHIDI	Korea Health Industry Development Institute
MAS	Modified Ashworth Scale
NIH	National Institute of Health
PP	per protocol
WUGMC	Wonkwang University Gwangju Medical Center
